# Selective parenchymal clamping in open partial nephrectomy: Early experience with a low cost, reusable novel parenchymal clamp

**DOI:** 10.1002/bco2.171

**Published:** 2022-06-23

**Authors:** James Macneil, Shuo Liu, Ramiz Iqbal, Finlay Macneil

**Affiliations:** ^1^ Department of Urology Hornsby Hospital Sydney New South Wales Australia; ^2^ Faculty of Medicine and Health Sciences Macquarie University Sydney New South Wales Australia; ^3^ Macquarie University Hospital Sydney New South Wales Australia; ^4^ Department of Urology Gosford Hospital Gosford New South Wales Australia

**Keywords:** partial nephrectomy, small renal mass, warm ischaemia

## Abstract

**Objectives:**

The objective of this study is to present our initial experience with a novel parenchymal clamp (NPC) developed to allow partial nephrectomies (PN) to be performed without whole kidney ischaemia. We compare patients who underwent PN with the NPC with those undergoing standard PNs.

**Methods:**

The NPC applies pressure only to the portion of the parenchyma containing the small renal mass (≤3.5 cm) and interrupts regional blood flow.

A retrospective analysis was conducted on patients that underwent open PN within our unit. Minimum follow‐up was 12 months. Patient and disease characteristics, perioperative outcomes and renal function estimated Glomerular Filtration Rate (eGFR) were compared.

**Results:**

Data were collected on 63 patients, of whom 33 had their procedure performed with the NPC. Demographic characteristics and comorbidity profiles were not significantly different between groups (p between 1.0 and 0.08). RENAL nephrometry scores were 5.6 in the NPC group versus 6.2 in the standard PN group (*p =* 0.146).

Perioperative, operative and postoperative data did not show significant differences. There was no difference in the rates of Clavien‐Dindo III or above complications between the two groups (NPC 3/33 vs. standard PN 5/30, *p =* 0.416). There was also no statistically significant difference to changes in renal functional at 12 months (change −5.2 and −6.4, *p =* 0.712).

**Conclusions:**

Despite the limited sample size and follow‐up, this study establishes the safety of the NPC. In the future, we intend to perform a prospective study on the laparoscopic version.

## 1 INTRODUCTION

The widespread use of abdominal imaging has led to increased detection of asymptomatic small renal masses (SRMs) ≤3.5 cm. In the context of renal cell carcinoma, this phenomenon has contributed to a significant stage shift towards T1 disease.^1^, ^2^ Longer‐term preservation of renal function is a key consideration when determining treatment modality. Over the last decade, elective partial nephrectomy (PN) has evolved to become the standard of care for the majority of SRM undergoing operation due to excellent oncological outcome and its capacity for nephron sparing.^3^, ^4^, ^5^, ^6^

The goals of any PN are, as defined in the Brassetti Trifecta,^7^ to achieve complete tumour excision, avoid postoperative complications and minimise renal function impairment. PN, open and laparoscopic (either pure or robot‐assisted), is most commonly performed with arterial clamping to establish a relatively bloodless surgical field. It has been widely accepted that warm ischaemic time should be as short as possible and less than 30 min.^8^, ^9^, ^10^, ^11^ However, some recent studies have demonstrated that even following a warm ischaemic time of under 20 min, medium‐ to long‐term accelerated loss of renal function can still occur.^12^, ^13^

Where possible, PN performed without arterial clamping decreases the risk of long‐term renal functional impairment. A recent study of 629 patients with an estimated Glomerular Filtration Rate (eGFR) <60 undergoing PN demonstrated a 7.3‐fold decrease in the risk developing severe renal impairment for patients having their operation performed without clamping the artery.^14^ However, off‐clamp PN is often difficult due to reasons of tumours characteristics and surgical expertise.

An alternative approach is the selective application of regional ischaemia. Traditionally, this is achieved with the surgical assistant firmly grasping the renal parenchyma, while the primary surgeon quickly performs tumour excision and renorrhaphy.^8^ Disadvantages include the possibility of assistant fatigue and potential needle stick injury. This technique may also limit the ability of a consultant surgeon to teach a trainee performing key aspects of the procedure as their grip must be released to do so. Nonpolar lesions are also less amenable to this technique. Some surgeons use selective ligation of a regional artery when this segmental vessel is accessible.

As an alternative to manually clamping the parenchyma of the kidney, a number of clamps have been used, including the Doyen bowel clamp, the Kauffman clamp and the Satinski clamp.^15^, ^16^ However, the uptake of these clamps has been slow due to a number of limitations.

**FIGURE 1:**
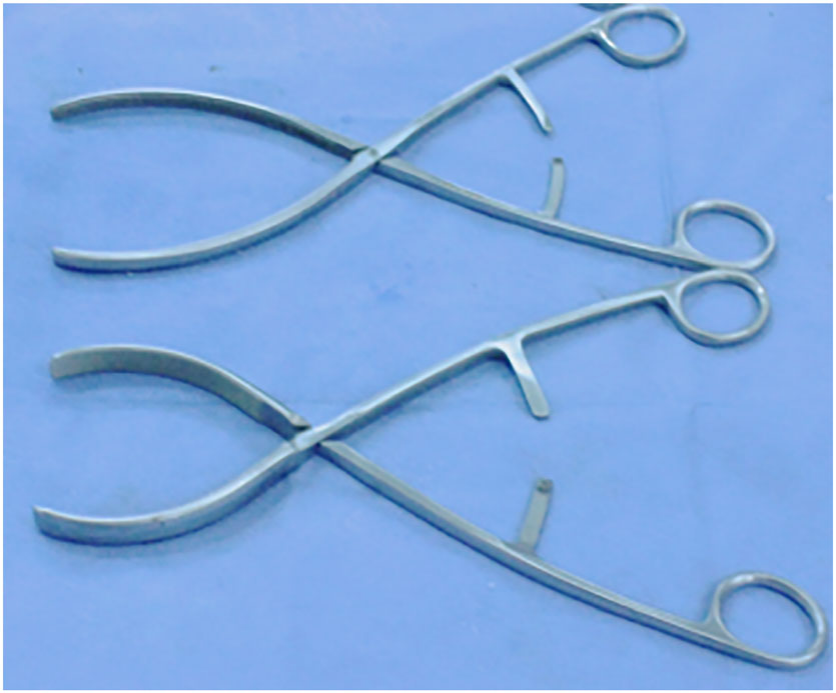
The first (top) and second (bottom) versions of the novel parenchymal clamp (NPC). Note the difference in shape to accommodate different contours of kidney parenchyma

**FIGURE 2:**
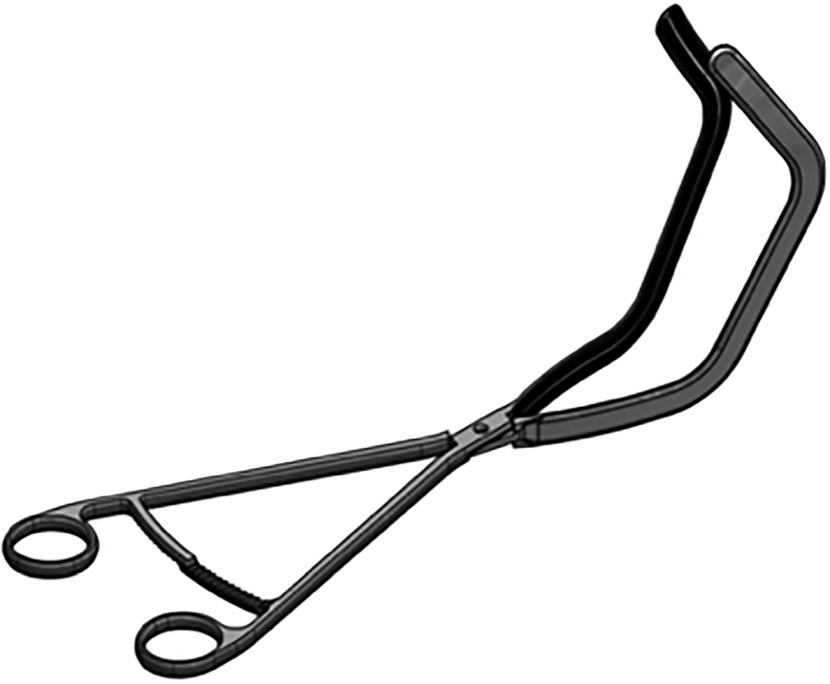
Diagram of the prototype of the third version of the novel parenchymal clamp (NPC)

**FIGURE 3:**
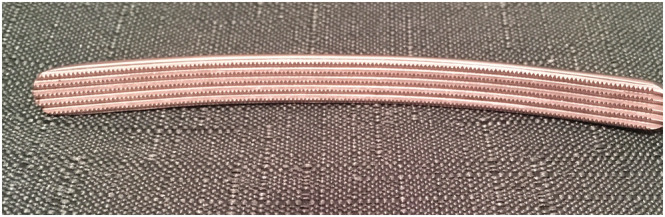
The milled teeth on the novel parenchymal clamp (NPC). These are common to all versions of the clamp

**FIGURE 4:**
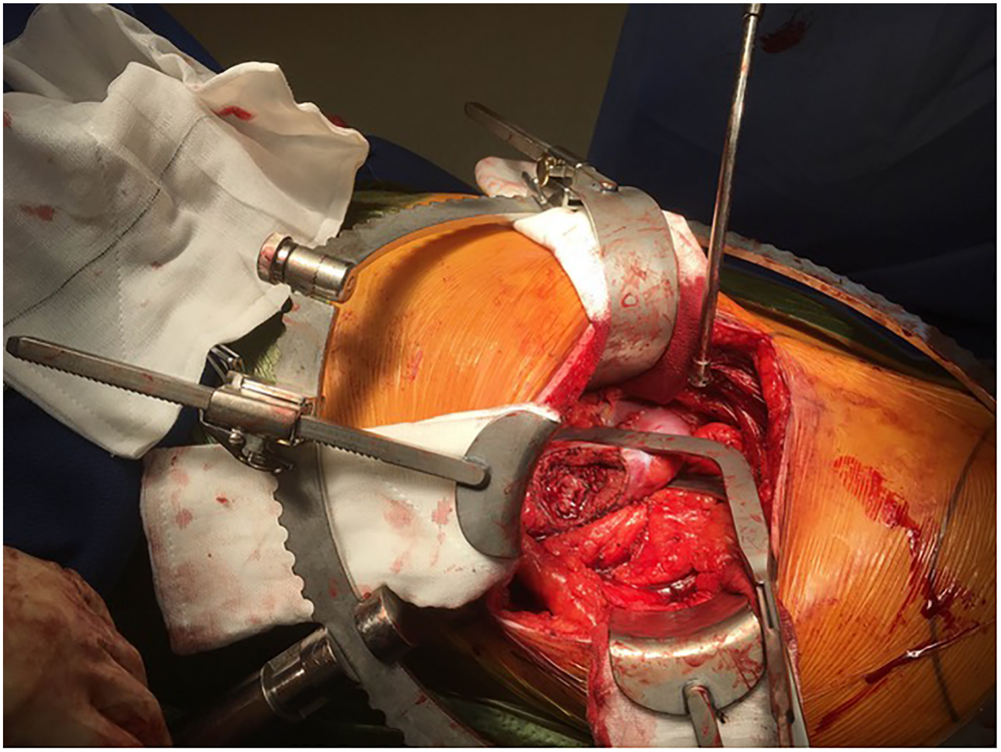
An application of the third version of the novel parenchymal clamp (NPC) to a nonpolar lesion without the hilum dissected

We have developed a set of novel parenchymal clamps (NPCs): the FinClamps I‐III (Figures 1, 2, 3, 4) for the purpose of open PN based on the principle of parenchymal compression and regional ischaemia. These clamps are specifically designed to obviate the need for hilar clamping as well as to overcome the shortcomings of assistant manual compression. This study reports our early experience with these novel clamps to define their safety and efficacy in open PN.

## 2 PATIENTS AND METHODS

This is a retrospective cohort study. We conducted an audit of all patients that underwent open partial nephrectomy (OPN) using the NPCs at our institution by the three contributing surgeons who now use the NPC during the development of the clamps. We compared these to those who had OPN with hilar clamping (the ‘standard’ technique, non‐NPC) during the same period. A total of three consultant urologists contributed to this series; patient selection was based on the discretion of individual surgeons. Each of the surgeons contributing patients to this series had been in practice for over 10 years at the start of the study period and was regularly performing partial nephrectomies,

A fourth surgeon who was also working in our institution during this period was periodically performing laparoscopic partial nephrectomies on appropriately selected patients. These laparoscopic cases were not included in the analysis to minimise the risk of introducing an unpredictable selection bias into the study.

A minimum follow‐up of 12 months was required for inclusion in the study. Patients with solitary kidneys, multiple unilateral renal lesions and those that required intra‐operative renal cooling were excluded—these cases were associated with greater surgical and medical complexity, and their postoperative renal functional recovery frequently followed a different course to those who had elective OPN.

### 2.1 The ‘standard’ OPN

In our institution, OPNs are typically conducted with flank incision into the retroperitoneal space and vascular clamping followed by tumour excision. We do not routinely perform renal cooling or administer intravenous mannitol and furosemide for single lesions. Basic haemostasis is achieved using a combination of sutures and electrocautery. Any defect in the collecting system is repaired. Renorrhaphy is performed with bolsters when primary closure is difficult. Haemostatic agents (such as Floseal®, Baxter, Deerfield IL USA) are applied occasionally.

### 2.2 OPN with NPC

The NPC was designed to apply just enough pressure (between 120 and 200 mmHg) to interrupt arterial flow only to the portion of the kidney containing the lesion. Compared with other parenchymal clamps, the curved jaws conform to the shape of the kidney and apply this pressure evenly. Figure 3 shows the milled teeth enabling the clamp to grip the renal capsule without slippage while avoiding cutting into the capsule and traumatising the tissue underneath. The ratchet mechanism enables incremental increase in pressure. This method allows the surgeon to perform meticulous and unrushed suturing and haemostasis before releasing the clamp.

The first and the second versions of the NPC are intended for removing polar lesions: variations in jaws accommodate for different positions of the tumour within the kidney as well as accounting for differences in renal contours (Figure 1). The exaggerated angled jaws of third version of the NPC enable the excision of more central lesions (Figure 2). This contrasts with other parenchymal clamps such as the Kauffman clamp in which the straight jaws of the clamp allow for little individualisation. Figure 4 demonstrates the use of the third version of the NPC in the excision of a nonpolar lesion.

In the early stage of the introduction of this technique, hilar dissection was regularly performed to permit rapid control of the vascular pedicle should the need arise. With increasing experience and confidence of individual surgeons using these devices, no hilar dissection was performed in later cases. The subsequent steps of the operation were the same as “standard OPN.

The NPC has been approved for use in Australia by the Therapeutic Goods Administration, and was approved by our institution prior to use. Patients were specifically consented prior to undergoing PN with the NPC. This study was conducted following review by the Hunter New England Human Research Ethics Committee, with approval given under reference number 17/09/20/5.12.

### 2.3 Data collection and analysis

Data were extracted from prospectively collected patient medical records, deidentified and transcribed into an electronic database. Demographic and background data including age, body mass index (BMI), American Society of Anesthesiology (ASA) score, previous surgery as well as common and major comorbidities were recorded. Perioperative data acquired included pre‐operative RENAL nephrometry score,^17^ estimated blood loss (EBL), transfusion rate, length of stay (LOS), complication and positive surgical margin rates. Baseline serum eGFR was measured within 2 weeks before PN. Postoperative eGFR was assessed at 3, 6 and 12 months. Statistical analysis was performed using SPSS version 20.0 (IBM, Armonk NY USA). Continuous variables were compared using an independent sample *t*‐test. Ordinal variables were analysed using either Pearson's chi‐squared test with continuity correction except where otherwise stated. A significant difference was defined as *p* < 0.05.

### 2.4 End points and power calculations

The primary end point of this study was the rate of Clavien‐Dindo III or above complications. Secondary endpoints include operative margin status and renal function at 6 and 12 months.

Power calculations were done using Cohen's technique,^18^ performed with the pwr package version 1.2‐2 by Stéphane Champlely et al. running in RStudio version 1.0.136 (RStudio Inc, Boston MA USA). Using the standard thresholds for effect size in Cohen's technique, to detect a small difference between the groups with 80% power (i.e. W = 0.1), we would need 385 patients in each arm. This was not deemed to be feasible in a single unit. For the sample size that we have, to detect medium differences (W = 0.3), our study had a power of 0.60–0.69.

## 3 RESULTS

From April 2012 to August 2017, a total of 63 patients had OPN performed for single tumours at our institution. Thirty‐three had their procedures performed using one of the NPCs. There were no conversions to radical nephrectomy. The mean age was 62.7 and the average BMI 29.6. Four had a pre‐operatively estimated GFR of less than 60 ml/min/1.73 m^2^: One had standard OPN, while the remainder 3 had their PN performed with the NPC.

Table 1 shows a comparison of baseline patient characteristics between the NPC and the standard OPN arms. There was no significant difference between the groups (*p* between 1.0 and 0.08).

**TABLE 1:** Baseline patient demographics

	NPC	Standard OPN	Difference
*n*	33	30	
Age in years (95% confidence interval)	65.3 (61.6–68.6)	59.9 (54.8–64.8)	t_61df_ = 1.763, *p =* 0.08
BMI (95% confidence interval)	29.3 (27.3–31.6)	30.0 (28.1–32.1)	t_61df_ = −0.414, *p =* 0.681
ASA (95% confidence interval)	2.3 (2.0–2.6)	2.2 (1.9–2.5)	t_59df_ = −0.690, *p =* 0.493
**Comorbidities**			
IHD	7/33	6/29^a^	*χ* ^2^_1df_ = 0.003, *p =* 1.000
Atrial fibrillation	2/33	1/30	*χ* ^2^_1df_ = 0.000, *p =* 1.000
Peripheral vascular disease	3/33	2/30	*χ* ^2^_1df_ = 0.000, *p =* 1.000
COPD	4/33	4/29^a^	*χ* ^2^_1df_ = 0.049, *p =* 1.000
Pre‐operative eGFR (range)	76.8 (71.3–81.8)	81.8 (75.8–87.5)	t_59df_ = −1.222, *p =* 0.227
Hypertension	5/33	4/30	*χ* ^2^_1df_ = 0.000. *p =* 1.000
Diabetes	2/33	6/29^a^	*χ* ^2^_1df_ = 2.939, *p =* 0.131
Insulin requirement	0/33	3/29^a^	*χ* ^2^_1df_ = 3.587, *p =* 0.097
Current smoker	5/33	8/29^a^	*χ* ^2^_2df_ = 1.714, *p =* 0.424^b^
Ex‐smoker	10/33	6/29^a^	
Regular immunosuppression	0/33	1/29^a^	*χ* ^2^_1df_ = 1.157, *p =* 0.468
Regular anticoagulation/platelet	8/33	4/29^a^	*χ* ^2^_2df_ = 1.080, *p =* 0.583^b^
Pre‐operative anticoagulation/platelet	1/33	1/29^a^	
Previous abdominal surgery	11/33	9/30	*χ* ^2^_1df_ = 0.000, *p =* 0.794

Abbreviations: ASA, American Society of Anesthesiology score; BMI, body mass index; COPD, Chronic Obstructive Pulmonary Disease; IHD, Ischaemic Heart Disease; NPC, novel parenchymal clamp.

^a^ Pre‐operative anaesthetic assessment could not be found for one patient, and full medical history could not be assessed for all parameters.

^b^ Pearson chi‐square without continuity correction (i.e. non‐2 × 2 table). *p*‐value is exact two‐sided significance.

As shown in Table 2, the average RENAL nephrometry score was 5.9 and the mean maximum diameter was 29.5 mm (range: 13–58 mm). Polar lesions were more likely to be encountered in the NPC arm (26 vs. 14, *p =* 0.016); otherwise, there were no significant differences.

**TABLE 2:** Tumour characteristics

	NPC	Standard OPN	Difference
Side	Left: 14 Right: 19	Left: 16 Right: 14	*χ* ^2^_1df_ = 0.454, *p =* 0.454
RENAL score (95% confidence interval)	5.6 (5.0–6.1)	6.2 (5.6–6.8)	t _61df_ = 1.474, *p =* 0.146
Maximum diameter (95% confidence interval)	2.9 (2.6–3.2)	3.0 (2.6–3.4)	t_60df_ = −0.422, *p =* 0.674
Diameter category	<=4 cm: 29 4‐7 cm: 4	<=4 cm: 25 4‐7 cm: 5	*χ* ^2^_1df_ = 0.024, *p =* 0.725
Exophytic proportion	> = 50%: 19 <50%: 11 Entirely endophytic: 3	> = 50%: 14 <50%: 9 Entirely endophytic: 7	*χ* ^2^_2df_ = 2.42, *p =* 0.298^a^
Proximity to collecting system	> = 7 mm: 18 4‐7 mm: 10 <=4 mm: 5	> = 7 mm: 16 4‐7 mm: 10 <=4 mm: 4	*χ* ^2^_2df_ = 0.086, *p =* 0.958^a^
Anterior/posterior split	Anterior: 15 Posterior: 12 Neither: 6	Anterior: 11 Posterior: 8 Neither: 11	*χ* ^2^_2df_ = 2.749, *p =* 0.253
Location relative to polar lines	Entirely above or below: 26 Lesion crosses polar lines: 4 >50% of mass crosses polar lines: 3	Entirely above or below: 14 Lesion crosses polar lines: 13 >50% of mass crosses polar lines: 3	*χ* ^2^_2df_ = 8.241, *p =* 0.016^a^

Abbreviations: NPC, novel parenchymal clamp; OPN, open partial nephrectomy.

^a^ Pearson Chi‐square without continuity correction (i.e. non‐2 × 2 table). *p*‐value is exact two‐sided significance.

Table 3 shows that although operative time, EBL, transfusion rates and LOS were lower with the use of NPC; none of these differences reached statistical significance. The same was also true for the incidence of urinary leaks (*p =* 0.34). Overall, there was no difference in the rates of Clavien‐Dindo III and above complications (3/33 for NPC versus 5/30, *p =* 0.416). One patient in each arm had a positive surgical margin. One postoperative death was observed in the NPC arm. The death occurred on postoperative day five due to sudden cardiac arrest. Autopsy did not establish any association between the cardiac event and the patient's operation.

**TABLE 3:** Comparison of perioperative outcomes

	NPC	Standard OPN	Difference
Operative time in minutes (95% confidence interval)	196 (176.6–214.4)	219 (203.2–235.3)	t_59df_ = 1.836, *p =* 0.071
Estimated blood loss in ml (95% confidence interval)	446 (366.9–525.7)	516 (409.0–623.5)	t_45df_ = 1.051, *p =* 0.299
Postoperative transfusion	1/33	2/30	*χ* ^2^_1df_ = 0.007, *p =* 0.601
Admission to ICU	1/33	2/30	*χ* ^2^_1df_ = 0.007, *p =* 0.601
LOS in days (95% confidence interval)	6.9 (5.9–8.1)	9.2 (6.2–12.7)	t_61df_ = 1.353, *p =* 0.181
Unplanned readmissions within 28 days	1/33	2/30	*χ* ^2^_1df_ = 0.007, *p =* 0.601
Complications by Clavien‐Dindo grade	‐I: 2 ‐II: 2 ‐IIIb: 1 ‐IVa: 1 ‐IVb: 0 ‐V: 1	‐I: 1 ‐II: 3 ‐IIIb: 3 ‐IVa: 2 ‐IVb: 0 ‐V: 0	*χ* ^2^_6df_ = 3.263, *p =* 0.659^a^
Urine leak	1/33	3/30	*χ* ^2^_1df_ = 0.379, *p =* 0.340
Positive margins	1/33	1/30	*χ* ^2^_1df_ = 0.005, *p =* 0.9454
Tumour type			*χ* ^2^_2df_ = 1.52, *p =* 0.468
Clear cell RCC	14	16	
Nonclear cell RCC	14	8	
Oncocytoma	5	2	
Other	0	4	

Abbreviations: LOS, length of stay; NPC, novel parenchymal clamp; OPN, open partial nephrectomy; RCC, Renal Cell Carcinoma.

^a^ Pearson Chi‐square without continuity correction (i.e. non‐2 × 2 table). *p‐*value is exact two‐sided significance.

The average warm ischaemic time for the standard PN arm was 23.2 min (range: 19–28); by the nature of the technique, the use of the NPC resulted in no warm ischaemia time. No significant difference was observed between groups in changes to eGFR from baseline renal function at 6 and 12 months (Table 4).

**TABLE 4:** Comparison of medium term postoperative eGFR

	NPC	Standard OPN	Difference
Change in eGFR at 6 months in ml/min/1.73m^2^ (95% confidence interval)	−1.1 (−5.9–9.1)	−5.6 (1.3–10.4)	t_36df_ = 1.068, *p =* 0.293
Change in eGFR at 6 months as a percentage of baseline (95% confidence interval)	−0.1% (−7.7–9.0)	−6.9% (1.4–13.2)	t_36df_ = 1.33, *p =* 0.192
Change in eGFR at 12 months in ml/min/1.73m^2^ (95% confidence interval)	−5.2 (−10.9 to −0.5)	−6.4 (−10.3 to −2.56)	t_36df_ = 0.371, *p =* 0.712
Change in eGFR at 12 months as a percentage of baseline (95% confidence interval)	−5.3% (−12.2 to −1.6)	−8.7% (−14.6 to −2.9)	t_38df_ = 0.726, *p =* 0.472

Abbreviations: NPC, novel parenchymal clamp; OPN, open partial nephrectomy.

## 4 DISCUSSION

The management of SRMs represents the intersection of two competing interests: the need to achieve sound oncological outcome as well as the preservation of functioning nephrons. The desire to maximise the latter has driven innovation and advances in the techniques for PN.

Many published series on PN demonstrate a median clamp time of about 20 min. When viewed in light of the recent evidence of late nephron loss with warm ischaemia times more than 20 min,^12^, ^13^ the need for a more physiological technique to achieve the objective of nephron preservation is evident. This is particularly relevant given the rise of chronic renal impairment in the community.^19^ Evidence of the response to this imperative can be found within the field of minimally invasive surgery (i.e. laparoscopic and robotically assisted PN) where there have been efforts to minimise the functional damage caused by PN via novel advancements in the technique (e.g. off‐clamp, selective arterial clamping and permissive hypotension),^20^, ^21^, ^22^ As mentioned previously, a variety of parenchymal clamps have been demonstrated in OPN previously; however, uptake has been comparatively poor.

This study demonstrates an alternative technique to manual clamping of the parenchyma with the advantage of being less prone to muscular fatigue, less operator dependent and less risk for needle stick injury. We believe that this will be crucially important in the future as progressively fewer urologists possess the depth of experience that preceding generations of urologists have had in complex open surgery.^23^

An additional advantage of this technique over hilar clamping is that it obviates the need for routine hilar dissection. This approach minimises the attendant scarring around the renal hilum and enables safer dissection of the renal vessels in the future if needed. This may account for the reduction in operative time in the NPC arm (although the difference in operative time between groups failed to achieve significance).

Although not yet commercially available, we anticipate a relatively low cost for the application of this technique as the sole additional requirement over a standard OPN is a set of reusable clamps. As compared with some of the techniques used to minimise renal parenchymal damage developed for use with minimally invasive surgery, we anticipate that the NPC could be of great utility in resource constrained settings.

Finally and very importantly is the facility of teaching trainees the techniques of PN. The NPCs free the hands of the teaching surgeon to demonstrate and guide the hands of the trainee without extending global warm ischaemia time or causing an increase in blood loss. Open surgical skills should be a crucial part of any upper tract surgeon's armamentarium even in the era of minimally invasive PN.^23^

The most obvious constraint of this study arises from it being a single institution study with a limited sample size, limiting what can be proved statistically (including the 8% difference in the rates of Clavien‐Dindo III and above complications.

There is also potentially a selection bias in favour of the NPC arm owing to the higher rates of of polar tumours in that cohort as polar lesions may be somewhat easier to excise with minimal parenchymal loss. The disparity is owing to the nonrandomised nature of this study as the NPC is easier to use on polar lesions. This potentially introduces a small advantage into the NPC arm with regard to renal function, though the degree can only be speculated upon. We do not believe that it will have impacted upon the rate of complications, and overall, we do not believe it invalidates our findings.

The other major limitation of this study is its retrospective, nonrandomised design. As demonstrated in Table 1, the groups are broadly comparable. Table 2 demonstrates that differences in the RENAL scores for standard versus NPC are neither statistically nor clinically different.

The key issue, however, is the effect of the NPC on renal function over an extended period of time. From the current study, we can say that there is no obvious difference between the groups within the first 12 months. However, the natural history of changes in renal function following renal surgery is measured in years, not months, as indeed are changes in all‐cause and cancer‐specific survival^24^ (which precludes this study from considering Cancer Specific Survival [CSS]).

Even given these limitations, this study is adequate for the purpose of demonstrating the feasibility and safety of our technique. We believe that it builds upon the works of previous studies of parenchymal clamping not only by having a substantially larger patient cohort but by including a direct comparison to patients undergoing standard OPN. We believe that the physiological rationale for the advantage of this technique should be apparent and that this study gives an adequate basis with which to consent a patient for the use of this novel technique.

At the time of writing, a laparoscopic a version of the clamp is under development. This instrument is being developed to be capable of deployment with little projection beyond the kidney to allow the kidney to be manipulated laparoscopically while maintaining haemostatic pressure on the parenchyma. We intend to study the utility of this clamp prospectively.

## 5 CONCLUSIONS

This study has demonstrated that parenchymal clamping with the NPC for OPN is a safe and effective technique. Our NPC enables partial nephrectomies without global warm ischaemia. It also facilitates effective teaching of the technique to trainees in a safe and nontime pressured environment. Although the present study is inadequate to prove superiority of the technique, it does show equivalence at 12 months.

Based on the safety of the technique as demonstrated by this study and our experience, we plan to further develop prospectively study the clamp and the technique with a laparoscopic version.

## CONFLICT OF INTEREST

FM declares a proprietary interest in the NPC as the designer and patent holder. JM, ML and RI declare no conflicts of interest.

## AUTHOR CONTRIBUTION

FM and ML contributed to study conception. JM, ML, and ML contributed to study design. RI and FM contributed to data collection. JM performed data analysis. ML, JM, and FM contributed to the preparation of the manuscript.
